# Relationship between the ST-Segment Resolution and Microvascular Dysfunction in Patients Who Underwent Primary Percutaneous Coronary Intervention

**DOI:** 10.1155/2019/8695065

**Published:** 2019-08-01

**Authors:** Byung Gyu Kim, Sung Woo Cho, Jeong-Ha Ha, Hyo Seung Ahn, Hye Young Lee, Gwang Sil Kim, Young Sup Byun, Kun Joo Rhee, Jong Chun Nah, Byung Ok Kim

**Affiliations:** ^1^Division of Cardiology, Department of Internal Medicine, Inje University College of Medicine, Seoul Paik Hospital, Seoul, Republic of Korea; ^2^Division of Cardiology, Department of Internal Medicine, Inje University College of Medicine, Sanggye Paik Hospital, Seoul, Republic of Korea; ^3^Division of Cardiology, Department of Internal Medicine, Sahmyook Medical Center, Seoul, Republic of Korea

## Abstract

**Objectives:**

Incomplete ST-segment elevation resolution (STR) occasionally occurs despite successful revascularization of epicardial coronary artery after primary percutaneous coronary intervention (PPCI). The aim of this study was to evaluate the relationship between the degree of STR and the severity of microvascular dysfunction.

**Methods:**

A total of 73 consecutive patients with ST-segment elevation myocardial infarction (STEMI) who underwent successful PPCI were evaluated. Serial 12-lead electrocardiography was performed at baseline and at 90 minutes after PPCI. Microvascular dysfunction was assessed by index of microvascular resistance (IMR) immediately after PPCI.

**Results:**

Patients were classified into 2 groups: 50 patients with complete STR (STR ≥50%) and 23 patients with incomplete STR (STR <50%). The incomplete STR group had a higher IMR value and lower left ventricular ejection fraction (LVEF), compared with the complete STR group. The degree of STR was significantly correlated with IMR (*r* = −0.416, *P*=0.002) and LVEF (*r* = 0.300, *P*=0.011). These correlations were only observed in patients with left anterior descending artery (LAD) infarction but not observed in patients with non-LAD infarction. A cutoff IMR value was 27.3 for predicting incomplete STR after PPCI.

**Conclusion:**

Incomplete STR after PPCI in patients with STEMI reflects the presence of microvascular and left ventricular dysfunction, especially in patients with LAD infarction.

## 1. Introduction

A successful restoration of epicardial coronary artery blood flow after primary percutaneous coronary intervention (PPCI) for ST-segment elevation myocardial infarction (STEMI) does not always lead to adequate myocardial perfusion or optimal outcome. Prior studies have shown that microvascular obstruction (MVO) is present in up to 50% of patients with STEMI even after timely reperfusion by PPCI and independently associated with ventricular remodeling and adverse clinical outcomes [1–6].

MVO can be identified by cardiac magnetic resonance imaging (MRI), but it can be performed couple of days after PPCI. Therefore, cardiac MRI is not directly helpful in determining whether potential therapies should be applied during revascularization or at early post-PPCI period to reduce adverse effect of MVO. However, measurement of ST-segment changes using electrocardiography (ECG) can not only be easily performed immediately after PPCI but also reflect status of microvascular integrity, infarct size, and prognostic information beyond angiographic findings [7–9]. However, impact of ST-segment elevation resolution (STR) was not fully investigated in PPCI era, and data of comparison between STR and other techniques evaluating microvascular function are relatively lacking.

Recently, the index of microvascular resistance (IMR) has been proposed for evaluating the coronary microvascular circulation and providing reliable quantitative values in patients with stable angina [10] and STEMI at the time of PPCI [11–14]. In the present study, we evaluated the relationship between incomplete STR and microvascular dysfunction assessed by IMR, which was performed immediately after PPCI.

## 2. Methods

### 2.1. Patient Population

The study population included 73 consecutive hemodynamically stable patients with the first attack of STEMI who underwent PPCI at Inje University Sanggye-Paik Hospital, Seoul, Korea, between March 2012 and November 2013. The inclusion criteria were as follows: (1) chest pain lasting ≥30 minutes and resistant to nitrates; (2) presentation within 12 hours after symptom onset; (3) persistent ST-segment elevation ≥2 mm in ≥2 contiguous precordial leads or ≥1 mm in ≥2 limb leads on 12-lead electrocardiography (ECG), and elevation of serum creatine kinase-MB (CK-MB) or troponin-I levels to at least twice the upper limit of normal; (4) angiographic evidence of total occlusion that is thrombosis in myocardial infarction (TIMI) flow grade 0 or 1. The exclusion criteria were as follows: (1) prior myocardial infarction, defined by preexisting pathologic Q-wave; (2) non-ST elevation myocardial infarction; (3) left bundle branch block, right bundle branch block, ventricular pacing, or other significant arrhythmia; (4) hemodynamic instability requiring the use of intravenous inotropes or mechanical supports such as intra-aortic balloon pump or percutaneous cardiopulmonary support.

All enrolled patients were treated with successful PPCI (defined as <25% residual epicardial lesion) in achievement of door-to-balloon times of ≤90 minutes and TIMI flow grade ≥2 in final angiography. Prior to PPCI, all patients received aspirin 300 mg, clopidogrel 600 mg, and weight-adjusted heparin (70 units/kg). Use of aspiration thrombectomy catheters or adjunctive pharmacologies such as platelet glycoprotein IIb/IIIa receptor inhibitor was left to the discretion of the primary operator. The study protocol was approved by the Institutional Review Board at our institute (SG-IRB 2010-085), and all participants provided written informed consent.

### 2.2. Measurement of Microvascular Function

After successful primary stenting, IMR was measured in all patients. IMR was assessed using a coronary pressure/temperature sensor-tipped guidewire placed distally in the culprit lesion. Hyperemia was induced by 140 *μ*g/kg/minute of intravenous adenosine preceded by a 2 mL intracoronary bolus of 200 *μ*g nitrate. The mean aortic and distal coronary pressures were recorded during maximal hyperemia. IMR was calculated as the formula of distal coronary pressure multiplied by the mean transit time of a 3 mL bolus of saline at room temperature during maximal coronary hyperemia (mm Hg·s or U) [11, 14]. We also evaluated angiographical assessment including TIMI flow grade and TIMI myocardial perfusion grade (TMP grade) using the method previously described [6] from the final recorded images.

### 2.3. Electrocardiographic and Echocardiographic Analysis

For all patients, a standard 12-lead ECG was obtained at initial hospital presentation and at 90 minutes after PPCI. ST-segment elevation was measured 20 ms after the J-point. The sum of ST-segment elevations (sum STE) was measured in leads V1 through V6 for anterior wall infarctions and in leads I, aVL, II, III, aVF, and V4 through V6 for nonanterior wall infarctions. STR was represented by percentage of summed ST-segment reduction between baseline and post-PPCI. The sum of residual ST-segment elevation (residual STE) across all infarct-related leads on the 90-minute post-PPCI ECG was also evaluated. Patients were divided into 2 groups according to the amount of STR: (1) complete STR (≥50%) and (2) incomplete STR (<50%). The ECGs were analyzed by 2 investigators blinded to IMR and angiographic data.

Standard echocardiography was performed after PPCI in all patients. According to the recommendation of the American Society of Echocardiography, the left ventricle was divided into 16 segments [15] and each segment was scored with a semiquantitative scoring system as follows: normal = 1, hypokinesia = 2, akinesia = 3, dyskinesia = 4, and aneurysm = 5. Left ventricular regional wall motion score index (RWMSI) was derived as a sum of all scores for each segment divided by the body surface area. Left ventricular ejection fraction (LVEF) was measured by the biplane Simpson method.

### 2.4. Biochemical Markers and Clinical Outcomes

The biochemical test was performed to obtain complete blood cell count finding, serum level of cardiac enzyme, N-terminal pro-B-type natriuretic peptide, and high-sensitivity C-reactive protein at the emergency room. CK-MB and troponin-I levels were measured at admission, six hours later, and then twice daily over first 48 hours. Peak CK-MB and peak troponin-I were defined as the highest CK-MB and troponin-I measured. All patients were followed for over 3 years, either by clinic visit or by telephone interview. The major adverse cardiovascular event (MACE) in this study was a composite of cardiac death, nonfatal myocardial infarction, any revascularization, and cerebrovascular accident. Clinical events were defined according to the Academic Research Consortium [16].

### 2.5. Statistical Analysis

Continuous variables are reported as mean ± standard deviation or median and categorical variables as a percentage. Continuous variables were analyzed using Student's *t*-test or Mann–Whitney *U*-test, and categorical variables data were compared using the Pearson *χ*^2^ test or Fisher's exact test. Correlations between two continuous variables were performed using the Pearson correlation coefficient. The best cutoff value of IMR for predicting incomplete STR 90 minutes after PPCI was determined by receiver operating characteristics (ROC) analysis. Based on this cutoff value, the low and high IMR were determined. Log-rank and Kaplan-Meier tests were used to compare survival between low and high IMR groups. A multivariate regression model was constructed to assess predictors of high IMR after PPCI. The associated variables with *P* value < 0.1 from univariate analyses were entered into the model. These analyses were performed using the software program SPSS, version 23.0 (IBM Corp., Armonk, NY, USA). All tests were 2-sided, and the results were considered statistically significant at *P* < 0.05.

## 3. Results

### 3.1. Baseline, Angiographic, and Procedural Characteristics

Baseline characteristics of the patients according to the degree of STR are summarized in Table 1. Twenty-three patients (31.5%) had incomplete STR at 90 minutes after successful PPCI. Sum STE was statistically comparable between 2 groups although numeric height was greater in the complete STR group. On analysis of ECG, left anterior descending artery (LAD) infarction was more often in the incomplete STR group, whereas non-LAD infarction was more common in the complete STR group. Patients with incomplete STR had significantly lower LVEF and higher RWMSI than those with complete STR. In addition, the degree of STR was significantly correlated with LVEF (*r* = 0.300, *P*=0.011) as well as RWMSI (*r* = 0.339, *P*=0.004). However, there were no differences of peak CK-MB and peak troponin-I between 2 groups.

Consistent with ECG analysis, angiography showed that LAD was more likely to be an infarct-related artery in the incomplete STR group compared with the complete STR group. However, proportion of TMP grade 1 achievement in the final angiography was not different between 2 groups.

### 3.2. Relationship between the Degree of STR and IMR according to Infarct Territory

**Figure 1:**
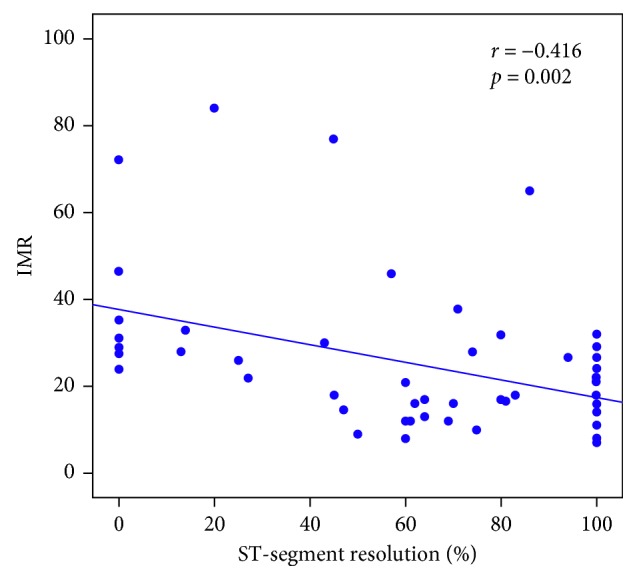
Relationship between STR and IMR. IMR: index of microvascular resistance; STR: ST-segment elevation resolution.

**Figure 2:**
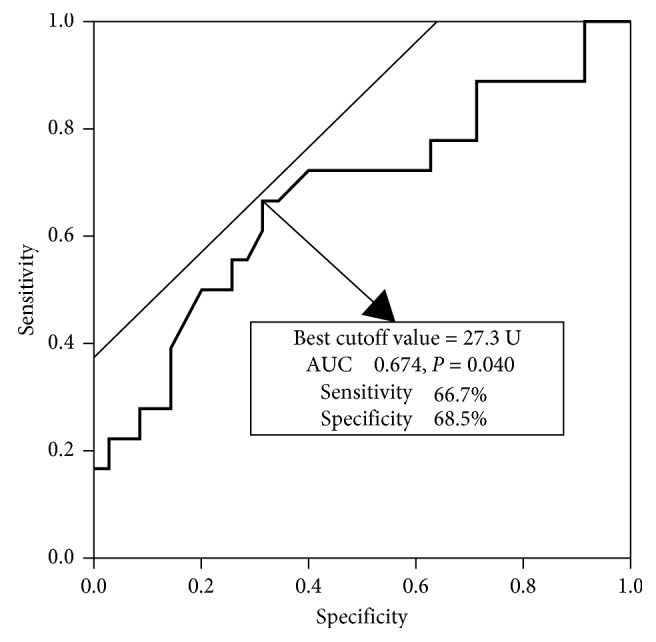
Receiver operator characteristic analysis to predict incomplete STR from the IMR value. The best cutoff value for IMR to predict incomplete STR was 27.3. AUC: area under the curve; IMR: index of microvascular resistance; STR: ST-segment elevation resolution.

**Figure 3:**
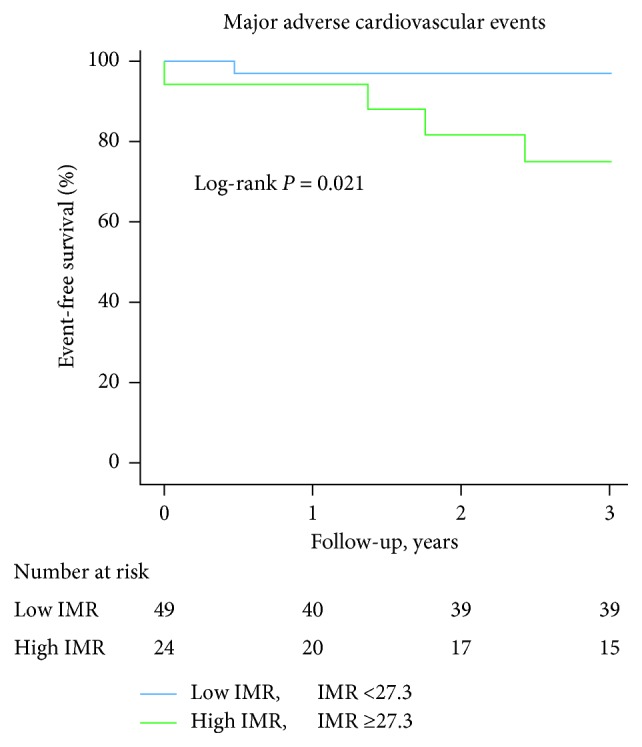
Kaplan–Meier curve between the high and low IMR group. IMR: index of microvascular resistance.

The IMR value was significantly higher in the incomplete STR group patients compared with the complete STR group (Table 1). In addition, a significant negative correlation was found between the degree of STR and IMR value (*r* = −0.416, *P*=0.002) (Figure 1). In patients with LAD infarction, those with incomplete STR had higher IMR and lower LVEF than those with complete STR (Table 2) and a significant negative correlation was observed between the degree of STR and IMR value (*r* = −0.411, *P*=0.020). In contrast, IMR and LVEF were not different between incomplete and complete STR groups, and no significant correlation was observed between the degree of STR and IMR value (*r* = −0.363, *P*=0.097) in patients with non-LAD infarction (Table 2). ROC analysis revealed a cutoff IMR value of 27.3 for predicting incomplete STR early after PPCI (Figure 2). When the patients were divided into the low and high IMR groups based on this cutoff value, incomplete STR was found to be an independent predictor of high IMR after PPCI in multivariate regression analysis (Table 3). Furthermore, the patients with high IMR had a higher incidence of MACE (16.6% vs. 2.0%, log rank *P*=0.021) than those with low IMR (Figure 3). However, incidence of MACE was not different between incomplete and complete STR groups (13.0% vs. 4.0%, log rank *P*=0.182).

### 3.3. Sum STE and Extent of Myocardial Damage

Despite the lack of a correlation of the degree of STR with the peak CK-MB and peak TnI, a strong relationship was identified between sum STE and the peak CK-MB (*r* = 0.346, *P*=0.003) and the peak TnI (*r* = 0.357, *P*=0.002). But, no correlation was found between the sum STE and IMR (*r* = 0.167, *P*=0.236).

## 4. Discussion

The present study has shown that the lack of STR early after STEMI treated with successful PPCI is linked with microvascular dysfunction assessed by IMR and left ventricular systolic dysfunction. To the best of our knowledge, this is the first study to demonstrate the association between STR and IMR after PPCI. Furthermore, the significant relationship between incomplete STR and higher IMR was observed in patients with LAD infarction, but not in those with non-LAD infarction. The cutoff value of IMR for predicting incomplete STR was 27.3, and patients with high IMR based on this value experienced more frequent incidence of MACE than those with low IMR.

Consistent with other studies [9, 17], our data showed that 31.5% of STEMI patients had incomplete STR after PPCI despite all achievement of TIMI 2 or 3 flow restoration. In these patients, impaired microvascular perfusion has been a plausible mechanism of persistent ST elevation due to ongoing metabolic derangement in the myocardial cellular level. In line with this hypothesis, some prior studies have reported that incomplete STR after PPCI was correlated with MVO assessed by positron emission tomography [13] and cardiac MRI [18]. Identification of MVO is crucial because patients with MVO are at higher risk for poor left ventricular function recovery, large infarct size, and adverse clinical outcomes [6, 19, 20]. However, those methods are not routinely performed in real-world practices. Furthermore, they cannot help us determine intra or early postinterventional treatment strategies because they can usually be performed at least 2 days after PPCI in medically stabilized patients. We established a relation between STR and IMR value providing the earliest quantitative assessment of microvascular status after PPCI. IMR can be readily obtained in the catheterization laboratory taking only a few minutes to measure and provides more consistent values than coronary flow reserve [21]. IMR has also confirmed its ability to predict extent of MVO, infarct size, and myocardial viability assessed by positron emission tomography [12] and cardiac MRI [14, 22]. Furthermore, previous studies have shown the impact of IMR for predicting adverse cardiovascular outcomes after PPCI [23, 24]. Fearon et al. firstly reported the value of IMR to predict left ventricular function recovery at 3 months after PPCI, but they found no linear correlation between the STR and IMR value [11]. But, their small population size might result in insufficient power to detect the relationship between STR and IMR. We demonstrated a good linear relationship between STR and IMR value with a cutoff value of 27.3 for predicting incomplete STR. Although the precise threshold of IMR related with MVO has not been elucidated yet, Carrick et al. recently presented IMR >27 was closely linked with the presence of MVO and myocardial hemorrhage on cardiac MRI [20], similar to our result. Furthermore, patients with IMR ≥27.3 experienced significantly higher incidence of MACE than those with IMR <27.3 in our study. Taken together, our findings suggest that the combined assessment of IMR and STR can be helpful to obtain more precise information about MVO and predict adverse outcomes in STEMI patients.

In the fibrinolysis era, STR was a well-established marker of the infarct-related artery (IRA) patency. Therefore, most of the previous fibrinolytic studies have shown the relation between a lack of STR and the large infarct size or poor clinical outcomes [25, 26]. However, there are insufficient data and some debate regarding the role and predictive value of STR after PPCI [27, 28]. Rakowski et al. showed that STR <70% in anterior wall MI was a marker of large infarct size assessed by both cardiac enzyme and cardiac MRI finding [27]. In this study, the degree of STR was not correlated with peak CK-MB of troponin-I which is regarded as a biochemical marker of the infarct size in this study. Instead, baseline sum STE showed a significant correlation with peak CK-MB and peak troponin-I. This can occurr because STR is the relative percentage of reduction from baseline sum STE. A marked difference in the severity of myocardial infarction might be present among patients who had similar STR according to whether patients were suffering from major or minor infarction. Our finding is consistent with previous data reported by Amaya et al. [13], suggesting the sum STE reflects the extent of myocardial infarction better than the degree of STR.

In the present study, patients with incomplete STR were more likely to have LAD infarction, similar to previous studies [9, 13, 29]. IMR was significantly higher and EF was lower in the incomplete STR than complete STR group in patients with LAD infarction. In patients with non-LAD infarction, however, IMR and EF were not different between the incomplete and complete STR group. These findings suggest that incomplete STR may be a more sensitive marker of microvascular and tissue injury in patients with LAD infarction than in those with non-LAD infarction. The exact mechanisms of poorer STR or microvascular reperfusion after PPCI in patients with LAD than non-LAD infarction are not fully investigated. Theoretically, the LAD usually supplies a larger territory of myocardium which may lead to more extensive myocardial injury at the microvascular level. Kandzari et al. demonstrated that LAD territory was associated with reduced left ventricular function, less frequent collateral flow, and impaired myocardial perfusion assessed by myocardial blush [30]. The existence of better collateral circulation in inferior versus anterior infarction could be a possible mechanism of similar IMR regardless of the degree of STR in non-LAD infarction in this study. When good collateral flow exists, it can augment distal coronary pressure and subsequently lead to an overestimation of IMR. However, we did not collect the data regarding existence of collateral flows in the present study. The results might be also due to the small study population. Therefore, further larger studies should be performed to identify whether intrinsic differences of microcirculatory perfusion are present between LAD and non-LAD infarction and clarify the applicable cutoff value of STR or IMR for predicting MVO in different infarct arteries.

According to our findings and previous studies, the degree of STR and IMR measurement may offer complementary information about microcirculatory dysfunction at the very earliest stage of STEMI management, so they may assist operator's early decision making to select patients who may benefit from adjunctive or more intensive therapy.

### 4.1. Limitations

The present study has several limitations. First, this was a prospective single-center observational study with a small study population. Second, we did not obtain follow-up echocardiography in all patients; therefore, we could not assess the interval change of ventricular function according to the degree of STR or IMR. At last, we excluded hemodynamically unstable patients who needed catecholamine or mechanical support because they could not be able to measure IMR. Therefore, only patients who had relatively small infarct area might be enrolled.

## 5. Conclusions

The degree of STR had an inverse relationship with the IMR value and was well correlated with LVEF. Incomplete STR early after PPCI for STEMI might be a surrogate marker for microvascular as well as left ventricular dysfunction, especially in patients with LAD infarction.

## Figures and Tables

**Table 1:** Baseline, angiographic, and procedural characteristics.

	All (*n*=73)	Incomplete STR (*n*=23)	Complete STR (*n*=50)	*P* value
Age (years)	58.4 ± 13.3	58.5 ± 13.3	58.4 ± 13.5	0.972
Male, *n* (%)	62 (84.9)	20 (87.0)	42 (84.0)	0.999
Comorbidities, *n* (%)				
Current smoker	37	13 (56.5)	24 (48.0)	0.618
Hypertension	41 (56.2)	13 (56.5)	28 (56.0)	0.967
Diabetes	16 (21.9)	5 (21.7)	11 (22.0)	0.980
Dyslipidemia	10 (13.7)	5 (21.7)	5 (10.0)	0.270
Stroke	6 (8.2)	1 (4.3)	5 (10.0)	0.658
Electrocardiographic data				
Infarct location, *n* (%)				
Anterior	33 (54.8)	17 (73.9)	23 (46.0)	0.042
Inferior	28 (38.4)	4 (17.4)	24 (48.0)	0.012
Lateral	5 (6.8)	2 (8.7)	3 (6.0)	0.672
Sum of ST-segment elevation (mm)	10.3 ± 5.6	9.2 ± 4.2	11.1 ± 6.0	0.137
ST-segment resolution (%)	63.8 ± 36.0	18.4 ± 22.8	84.7 ± 16.1	<0.001
Residual sum of ST-segment elevation (mm)	3.6 ± 3.5	6.6 ± 3.2	2.1 ± 2.6	<0.001
Laboratory data				
Absolute neutrophil count (mm^3^)	7,959 ± 3,797	8,704 ± 3,936	7,616 ± 3,722	0.259
Peak CK-MB (IU/L)	191.9 ± 130.7	215.8 ± 129.4	180.8 ± 131.1	0.291
Peak troponin-I (ng/ml)	52.0 ± 30.5	57.0 ± 29.1	49.7 ± 31.1	0.340
N-terminal pro-BNP (pg/ml)	585.3 ± 875.4	677.0 ± 999.6	527.9 ± 805.6	0.611
hsCRP (mg/L)	1.41 ± 2.56	2.0 ± 3.7	1.17 ± 1.92	0.368
Echocardiography finding				
Ejection fraction (%)	51.4 ± 10.9	45.5 ± 7.8	54.2 ± 11.1	0.001
Ejection fraction <50%, *n* (%)	32 (43.8)	17 (73.9)	15 (30.0)	0.001
RWMSI	1.32 ± 0.32	1.47 ± 0.35	1.25 ± 0.27	0.005
Angiographic data				
LAD culprit, *n* (%)	33 (54.8)	17 (73.9)	23 (46.0)	0.042
Coronary flow before PPCI, *n* (%)				
TIMI flow grade 0 or 1	54 (74.0)	18 (78.3)	36 (72.0)	0.775
TIMI flow grade 2 or 3	19 (26.0)	5 (21.7)	14 (28.0)	0.775
Coronary flow after PPCI, *n* (%)				
TIMI flow grade 2	15 (20.5)	7 (30.4)	8 (16.0)	0.213
TIMI flow grade 3	58 (79.5)	16 (69.6)	42 (84.0)	0.213
TMP grade after PPCI, *n* (%)				
TMP grade 1	2 (2.7)	1 (4.3)	1 (2.0)	0.534
TMP grade 2	29 (39.7)	11 (47.8)	18 (36)	0.337
TMP grade 3	42 (57.5)	11 (47.8)	31 (62)	0.255
Index of microvascular resistance (U)	24.3 ± 17.6	32.6 ± 23.5	20.0 ± 11.8	0.012
Procedure data				
Mean stent diameter (mm)	3.4 ± 0.5	3.3 ± 0.4	3.5 ± 0.5	0.217
Total stent length (mm)	21.7 ± 6.6	22.4 ± 7.8	21.3 ± 6.0	0.498
No reflow, *n* (%)	3 (4.1)	1 (4.3)	2 (4.0)	0.945
Aspiration thrombectomy, *n* (%)	66 (90.4)	21 (91.3)	45 (90.0)	0.999
Use of platelet glycoprotein IIb/IIIa inhibitors, *n* (%)	35 (47.9)	11 (47.8)	24 (48.0)	0.989

BNP: brain natriuretic peptide; CK-MB: creatine kinase-MB; hsCRP: high-sensitivity C-reactive protein; LAD: left anterior descending artery; MACE: major adverse cardiovascular event; PPCI: primary percutaneous coronary intervention; RWMSI: regional wall motion score index; STR: ST-segment elevation resolution; TIMI: thrombosis in myocardial infarction; TMP grade: TIMI myocardial perfusion grade.

**Table 2:** Comparison of LAD and non-LAD infarctions.

	LAD infarction (*n*=40)			Non-LAD infarction (*n*=33)		
	Incomplete STR (*n*=17)	Complete STR (*n*=23)	*P* value	Incomplete STR (*n*=6)	Complete STR (*n*=27)	*P* value
ST-segment resolution (%)	24.2 ± 18.4	78.1 ± 15.9	<0.001	29.5 ± 40.0	90.3 ± 14.3	0.013
Ejection fraction (%)	43.4 ± 5.0	51.3 ± 13.6	0.015	51.6 ± 11.5	56.8 ± 7.8	0.183
RWMSI	1.6 ± 0.4	1.4 ± 0.3	0.145	1.2 ± 0.2	1.1 ± 0.1	0.133
Peak CK-MB (IU/L)	236.8 ± 130.9	197.3 ± 147.0	0.382	156.3 ± 114.5	166.8 ± 116.9	0.843
Peak troponin-I (ng/ml)	60.5 ± 28.2	49.7 ± 34.5	0.281	47.2 ± 32.1	49.6 ± 28.5	0.852
TMP grade after PCI						
1, *n* (%)	1 (5.9)	0	0.425	0	1 (3.7)	0.818
2, *n* (%)	9 (52.9)	10 (43.5)	0.554	2 (33.3)	8 (29.6)	0.858
3, *n* (%)	7 (41.2)	13 (56.5)	0.337	4 (66.7)	18 (66.7)	0.999
Index of microvascular resistance (U)	33.7 ± 25.0	20.7 ± 14.4	0.046	28.8 ± 19.9	19.4 ± 8.7	0.148

CK-MB: creatine kinase-MB; LAD: left anterior descending artery; RWMSI: regional wall motion score index; STR: ST-segment elevation resolution; TMP grade: TIMI myocardial perfusion grade.

**Table 3:** Univariate and multivariate regression analyses for predicting high IMR (IMR ≥27.3).

	Univariate analysis		Multivariate analysis	
	HR (95% CI)	*P* value	HR (95% CI)	*P* value
Age, per year	1.01 (0.97–1.06)	0.545		
Male	0.76 (0.16–3.59)	0.727		
Hypertension	1.53 (0.50–4.75)	0.458		
Diabetes mellitus	1.25 (0.33–4.70)	0.741		
Dyslipidemia	1.70 (0.34–8.55)	0.520		
Current smoking	2.06 (0.65–6.51)	0.217		
Incomplete STR	4.03 (1.22–13.28)	0.022	4.80 (1.06–21.69)	0.042
Peak CK-MB	1.01 (1.00–1.01)	0.060	1.00 (1.00–1.01)	0.265
Peak troponin-I	1.02 (1.00–1.04)	0.070	1.01 (0.98–1.05)	0.534
hsCRP	1.05 (0.85–1.30)	0.659		
Ejection fraction	0.94 (0.87–1.01)	0.091	1.00 (0.91–1.11)	0.878
Aspiration thrombectomy	0.48 (0.07–3.44)	0.500		
Stent length	1.05 (0.96–1.15)	0.255		

CI: confidence interval; CK-MB: creatine kinase-MB; HR: hazard ratio; IMR: index of microvascular resistance; STR: ST-segment elevation resolution.

## Data Availability

The data used to support the findings of this study are available from the corresponding author upon request.
